# One-Step Engineering Carbon Supported Magnetite Nanoparticles Composite in a Submicron Pomegranate Configuration for Superior Lithium-Ion Storage

**DOI:** 10.3390/ma16010313

**Published:** 2022-12-29

**Authors:** Mengyao Tu, Chun Yang, Rui Zhang, Xiangli Kong, Ruixin Jia, Longbiao Yu, Binghui Xu

**Affiliations:** Institute of Materials for Energy and Environment, College of Materials Science and Engineering, Qingdao University, Qingdao 266071, China

**Keywords:** spray pyrolysis, chitosan, magnetite nanoparticle, pomegranate configuration, lithium-ion batteries

## Abstract

In this work, magnetite nanoparticles (Fe_3_O_4_) that are well dispersed by a submicron sized carbon framework in a pomegranate shape are engineered using a flexible one-step spray pyrolysis strategy. Under inert gas atmosphere, the homogeneously mixed Fe^3+^ ions and chitosan (CS) molecules are in situ transformed to Fe_3_O_4_ nanoparticles and spherical nitrogen-doped carbon coating domains, respectively. Moreover, the obtained Fe_3_O_4_@C composite exhibits a unique submicron sized pomegranate configuration, in which favorable electric/ionic pathways have been constructed and the Fe_3_O_4_ nanoparticles have been effectively dispersed. When used as an anode electrochemical active material, the Fe_3_O_4_@C composite exhibits impressive lithium-ion storage capabilities, and maintains a reversible capacity of 500.2 mAh·g^−1^ after 500 cycles at a high current density of 1000 mA·g^−1^ as well as good rate capability. The strategy in this work is straightforward and effective, and the synthesized Fe_3_O_4_@C material has good potential in wider applications.

## 1. Introduction

Due to the increasing demand for electric vehicles and various electronic devices, lithium-ion batteries with high energy density and high-rate performance are developing rapidly [1,2]. In 2000, Poizot et al. first reported the reversible lithium-ion storage capability of transition metal oxides (TMOs) as a potential anode material for lithium-ion batteries [3], and in the following two decades, the investigation of TMO-based materials have led to considerable concerns. Magnetite (Fe_3_O_4_) has an attractive theoretical capacity of 926 mAh·g^−1^ together with the benefits of cost-effectiveness, non-toxicity, and environmentally friendliness [4,5]. However, hampered by their inherent problems of low conductivity and severe volume expansion, there is still a large gap before the practical application of Fe_3_O_4_-based materials in commercial lithium-ion batteries [6,7]. The widely recognized strategies for properly solving the above problems of Fe_3_O_4_ are mainly divided into two categories: one is to construct nanostructures with a special morphology to alleviate the strain caused by lithiation/delithiation and shorten the lithium-ion transport pathways [8,9,10]; the other is to engineer a carbonaceous supportive frame to accommodate the volume change and thus maintain the integrity of the corresponding composite electrode [11,12,13,14].

Chitosan (CS) is the product from the natural polysaccharide chitin after partial removal of the acetyl groups, which is considered as a low cost, environmentally friendly material [15]. Moreover, the CS chain is rich in hydroxyl, amino and other functional groups, which enable CS to effectively interact with transition metal ions to form corresponding complex compounds [16]. Therefore, CS molecules are considered as one of the most ideal precursor materials in the synthesis of nitrogen-doped porous carbon-based materials for electrochemical storage. Shao and coworkers [17] selected CS as the nitrogen-containing carbon source, which was carbonized in a ZnCl_2_ molten salt at a temperature of 600 °C to prepare a porous carbon sample with the most optimal performance in supercapacitors. In another report, taking the advantages of the strong coordination between hydroxyl and amino groups of CS molecules and Fe^3+^ ions, a conformal and continuous CS derived nitrogen-doped carbon coating layer was spontaneously engineered on Fe_3_O_4_ particles by combining a 180 °C hydrothermal reaction and a further 600 °C calcination [18]. The as-prepared nanocomposites showed a dramatically enhanced lithium storage reaction kinetic with high specific capacity and outstanding rate capability. On the other hand, the aerosol spray pyrolysis technique is facile and effective in engineering a spherical carbon matrix with nano-sized electrochemically active materials that are uniformly dispersed [19]. The rapid heating of precursor droplets and the subsequent cooling of the product could significantly control the size of the electrochemically active component and prevent the excessive aggregation of the carbon skeletons. Therefore, a superior rate performance and cycling stability of the composite sample can be achieved. In our previous work, it was proven that the in situ engineering carbon framework and Fe_3_O_4_ crystals could produce significantly improved electric/ionic conductivity and effectively alleviated the aggregation of Fe_3_O_4_ nanoparticles [12,20]. Therefore, more inspiring lithium-ion storage performances have been delivered from the corresponding composite materials.

**Scheme 1:**
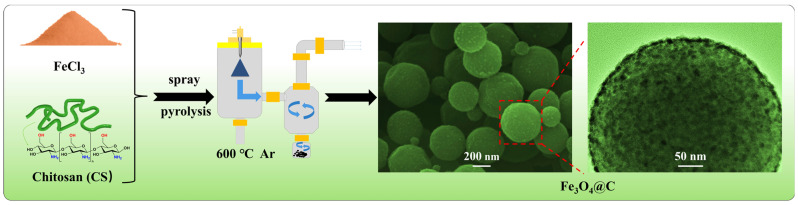
Schematic illustration for the synthesis of the Fe_3_O_4_@C sample.

On the above foundation, it is of great significance to explore simplified material synthesis methods and enhance the atomic efficiency for carbon supported nano-sized Fe_3_O_4_ composites. It is particularly interesting to jointly use CS molecules as carbon framework precursors and to employ spray pyrolysis treatment to engineer corresponding composite materials with an exceptional microstructure. In the present work, as shown in Scheme 1, a CS derived submicron sized carbon framework in a pomegranate shape has been engineered to disperse Fe_3_O_4_ nanoparticles with controlled sizes using a flexible one-step spray pyrolysis strategy. Protected by inert gas, the homogeneously mixed Fe^3+^ ions and CS molecules are in situ transformed to homogeneously dispersed Fe_3_O_4_ nanoparticles with controlled size and spherical pyrolytic carbon supporting domains with high mechanical strength, respectively. Moreover, favorable conductive networks and buffering space have been created in the Fe_3_O_4_@C composite. When used as an anode electrochemical active material, the Fe_3_O_4_@C composite exhibits inspiring lithium-ion storage capabilities, which maintains a high specific capacity of 865.0 mAh·g^−1^ at a low current density of 200 mA·g^−1^ after 120 cycles and a stable reversible capacity of 500.2 mAh·g^−1^ at a high current density of 1000 mA·g^−1^ after 500 cycles. The strategy in this work is straightforward and effective, and the synthesized Fe_3_O_4_@C material has good potential in wider applications.

## 2. Materials and Methods

### 2.1. Sample Synthesis

The chemicals are analytically pure and were purchased from the Sinopharm Company and used as received.

FeCl_3_·6H_2_O (1.0 g) was at first dissolved by a proper amount of deionized water. Next, CS (1.0 g) was gradually added to the above suspension. The system was maintained by stirring for 2 h. The above mixture underwent a spray pyrolysis treatment using an Ultrasonic Spray Pyrolysis System under an inert atmosphere at 600 °C to obtain the final black Fe_3_O_4_@C powder sample.

### 2.2. Sample Characterization and Electrochemical Measurement

An X-ray diffraction (XRD) analysis was performed using an X-ray diffractometer (Rigaku Ultima IV, Tokyo, Japan) with a Cu-Kα radiation of λ = 0.15418 nm, and the scanning speed was 10 °·min^−1^ with a grazing angle of 5°. A field-emission scanning electron microscope (FESEM, JEOL JSM-7800F, Tokyo, Japan) with an energy dispersive X-ray (EDS) accessory and a transmission electron microscope (TEM, JEOL JEM-2100Plus) were used to investigate the microstructures of the prepared samples. A Renishaw inVia Plus Micro-Raman spectroscopy system equipped with a 50 mW DPSS laser at 532 nm was used to record the Raman spectra of the samples. X-ray photoelectron spectroscopy (XPS) was measured using a PHI Quantera II spectrometer with compressed samples pasted on a testing platform. A thermogravimetric analysis (TGA) was carried out to determine the content of components by a thermal gravimetric analyzer (TGA-2, Mettler Toledo, Zurich, Switzerland) in air with a heating rate of 10 °C min^−1^ from 25 °C to 800 °C.

Coin-type CR2016 half cells were used to evaluate the lithium-ion storage performances of samples with metal lithium foil as a counter and reference electrode. Fe_3_O_4_@C, carbon fiber, acetylene black and polyvinylidene fluoride were mixed in a weight ratio of 7:1:1:1, then pasted on a copper foil and dried as a working electrode. A Celgard 2600 film was used as a separator between the two electrodes. A solution with 1 M LiPF6 dissolved in ethylene carbonate/dimethyl carbonate/ethylene methyl carbonate (EC/DMC/EMC) with a volumetric ratio of 1:1:1 was used as an electrolyte. 

Cyclic voltammetry (CV) measurements were performed on a CHI 660E electrochemical workstation at a scanning rate of 0.1 mV·s^−1^. Galvanostatic charge and discharge testing was carried out on a Neware battery test instrument, and the specific capacity was calculated based on the mass of the synthesized composite samples. The electrochemical impedance spectroscopy (EIS) testing was also carried out using the CHI 660E electrochemical workstation at a frequency range from 100 kHz to 1 Hz with a signal amplitude of 5 mV.

## 3. Results

### 3.1. Structure and Morphology

**Figure 1:**
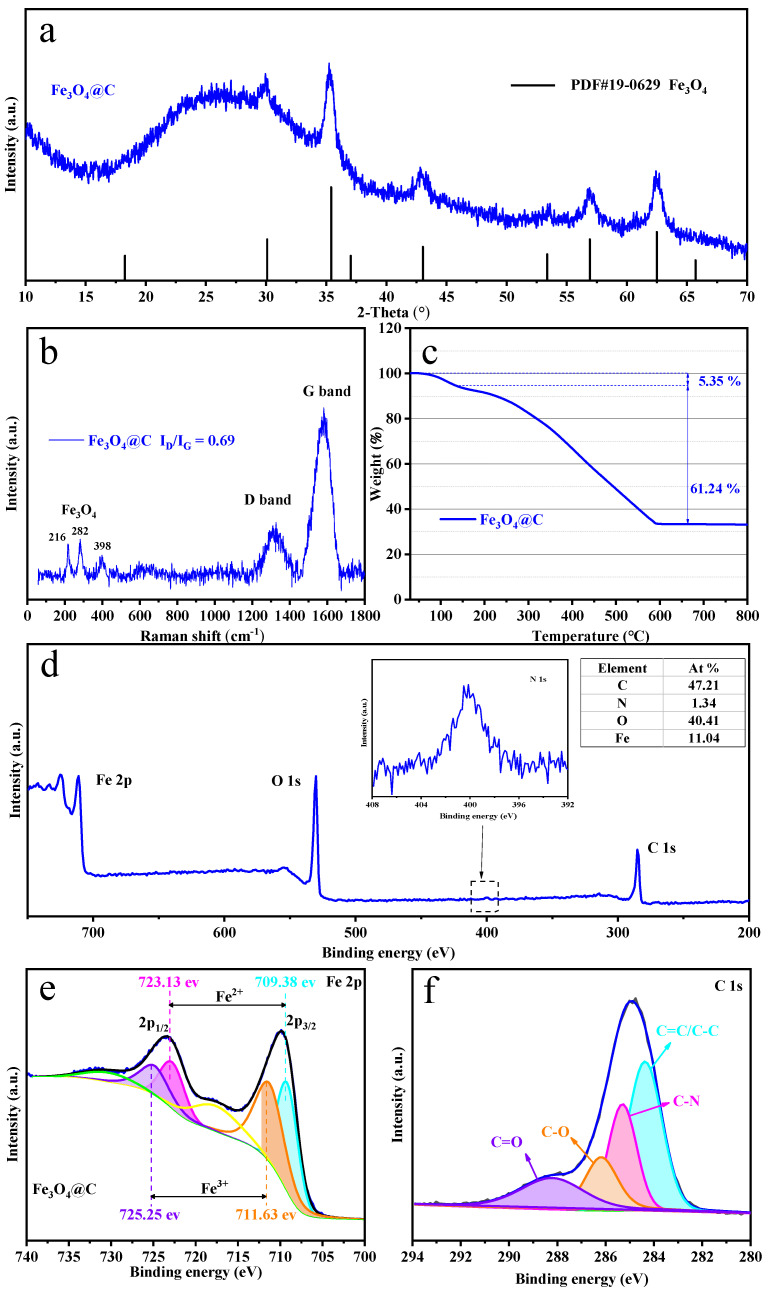
XRD pattern (**a**), Raman spectrum (**b**), TGA curve (**c**), survey XPS spectrum (**d**), high-resolution Fe 2p (**e**) and C 1s (**f**) XPS spectra of the Fe_3_O_4_@C sample.

From the XRD characterization results of the Fe_3_O_4_@C sample in Figure 1a, it can be seen that the distinct characteristic diffraction peaks at 30.1, 35.4, 43.1, 56.9 and 62.5° two theta are well matched to the (2 2 0), (3 1 1), (5 1 1) (4 4 0) crystal planes of Fe_3_O_4_. It can be speculated that the redox reaction has taken place between the Fe^3+^ ions and the CS molecules during the spray pyrolysis treatment. The broad peak at approximately 26° two theta indicates the formation of a carbon framework derived from CS molecules. 

From the Raman spectrum of the Fe_3_O_4_@C sample in Figure 1b, it can be seen that there are two obvious peaks around 1350 cm^−1^ (D band) and 1590 cm^−1^ (G band), which are related to the carbon plane defects and the in-plane expansion vibration of sp2 carbons, respectively [21,22]. Therefore, the peak-intensity ratio (I_D_/I_G_) of the D band to the G band reflects the defect degree from carbon materials [22]. The I_D_/I_G_ value for the Fe_3_O_4_@C sample was measured to be 0.69, indicating that the conductive conjugated carbon network has been formed from the pyrolysis of CS molecules. At the same time, small Raman peaks can be seen at 216, 282 and 398 cm^−1^ from the Fe_3_O_4_@C sample, which can be attributed to the Fe_3_O_4_ species.

Figure 1c shows the TGA curve of the Fe_3_O_4_@C sample in air. The initial weight loss from 30 °C to 150 °C is 5.35%, which is related to the evaporation of water. The quality degradation mainly occurs from 300 °C to 600 °C, where the decomposition of CS derived supporting framework and the conversion of immobilized Fe_3_O_4_ nanoparticles to Fe_2_O_3_ species have taken place. Based on the TGA curve, it is estimated that the Fe_3_O_4_ crystals occupy 32.28% of the total weight of the Fe_3_O_4_@C sample.

The measured XPS wide-scan spectrum of the Fe_3_O_4_@C sample is shown in Figure 1d, where C, O and Fe elements can be clearly identified while a weak N signal can also be detected in the inset locally magnified spectrum. Among them, the content of C and O elements is quite high, while the content of Fe element is relatively low. This result demonstrates that the engineered carbon skeleton is effective in immobilizing the Fe_3_O_4_ nanoparticles in the Fe_3_O_4_@C sample, while some oxygen-containing radicals still exist on the CS derived carbons. The high-resolution XPS spectrum of Fe 2p is shown in Figure 1e. It can be observed that the main peaks of the Fe_3_O_4_@C sample at 711.63 and 709.38 eV belong to typical Fe^3+^ and Fe^2+^, and the binding energies of Fe 2p1/2 and Fe 2p3/2 are 724.8 eV and 711.3 eV, respectively, corresponding to the spin orbital peak of Fe_3_O_4_ [23,24]. In addition, from the C 1s fine spectrum in Figure 1f, the four peaks with binding energies of 284.6, 285.4, 287.7 and 288.9 eV correspond to C=C/C-C (sp2/sp3), C-N, C-O (hydroxyl and epoxy) and O-C=O (carboxyl), respectively [25,26,27]. It can be seen that the C-C/C=C carbon bond in the composite is relatively strong among the divided peaks, which proves that the carbon skeleton has been constructed from the pyrolysis of CS molecules. The C-N bond indicates the successful introduction of N in the carbon lattice, which is widely recognized as being beneficial to improving the conductivity of the carbon network.

**Figure 2:**
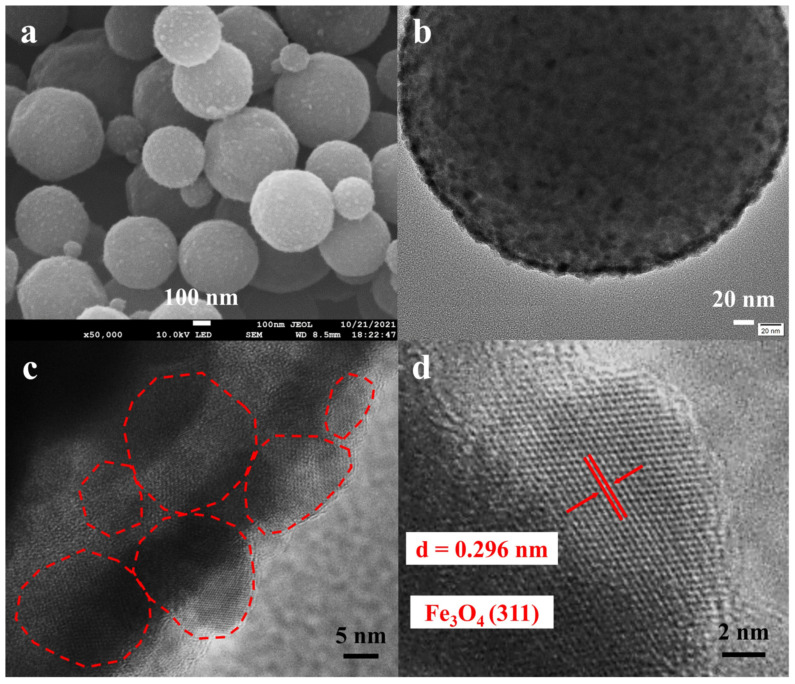
FESEM image (**a**), TEM images under different magnifications (**b**,**c**) and HRTEM image (**d**) of the Fe_3_O_4_@C sample.

Figure 2a shows the overall FESEM image of the Fe_3_O_4_@C composite, and it can be seen that the submicron sized spherical structures are without obvious structural damage. The TEM image of the individual Fe_3_O_4_@C composite is shown in Figure 2b. The homogeneously dispersed Fe_3_O_4_ nanoparticles and continuous carbon supporting matrix jointly form the unique pomegranate microstructure. From the locally magnified TEM image in Figure 2c, the Fe_3_O_4_ nanoparticles with controlled sizes marked by red dash lines are well distributed in the carbon domain, and space can be clearly seen between these and the Fe_3_O_4_ nanoparticles. According to the HRTEM image in Figure 2d, thin carbon coating layers can be identified on the Fe_3_O_4_ nanocrystal. Moreover, a clear lattice distance of 0.296 nm is a good reflection of the (3 1 1) orientation of Fe_3_O_4_ crystals, which matches well with the XRD and XPS characterization results.

**Figure 3:**
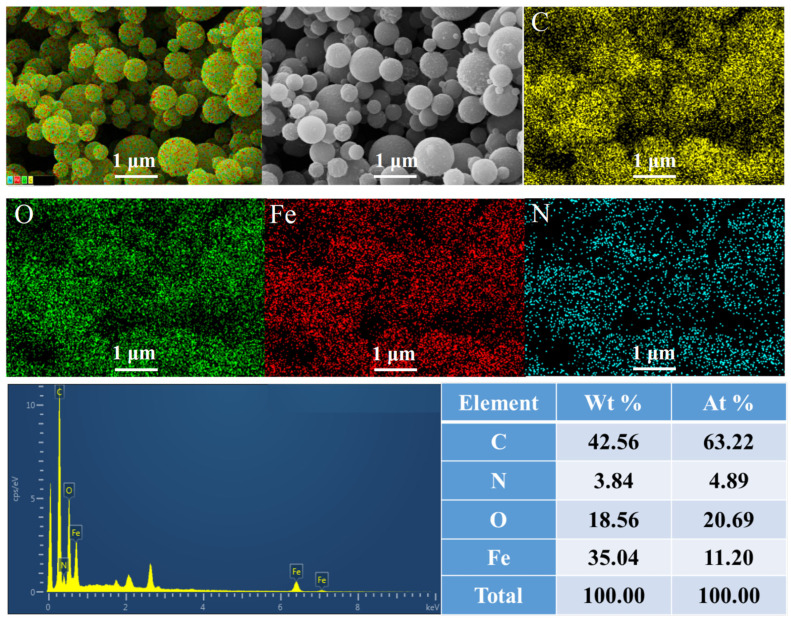
EDS mapping and element analysis result of the Fe_3_O_4_@C sample.

The EDS mapping and elemental analysis result of the Fe_3_O_4_@C sample are shown in Figure 3, where the C, O, Fe and N elements can be verified, and the atomic percentages of these elements are quite consistent with the XPS results. It can be seen that the distribution of the four elements is in good agreement with each other, which indicates the homogeneous dispersion of smaller sized Fe_3_O_4_ nanoparticles in the submicron sized spherical carbon supporting matrix.

### 3.2. Lithium-Ion Storage Performances

**Figure 4:**
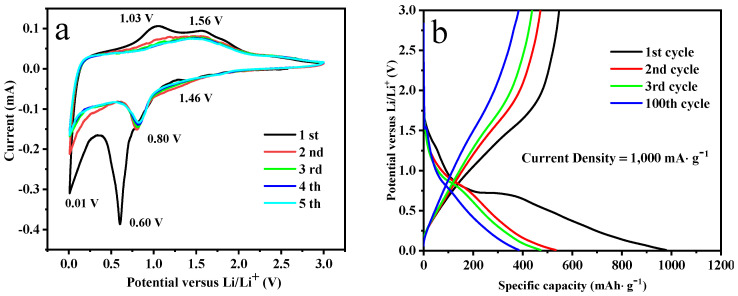
The CV curves for the initial five cycles (**a**), charge and discharge voltage profile (**b**) of the Fe_3_O_4_@C composite.

The CV curves of the Fe_3_O_4_@C composite are shown in Figure 4a. During the first discharge, unlike the following CV curves, a weak peak of about 1.46 V appears in the cathodic scan, and a significant cathode peak can be found at 0.6 V, which can be attributed to the reduction of Fe_3_O_4_ nanocrystals to generate metallic Fe phases and the formation of the solid-electrolyte interphase (SEI), respectively [28,29,30,31]. In subsequent cycles, there is only one significant peak of approximately 0.8 V from the cathodic section, which is due to the reversible reduction of iron oxide [32,33]. The first anodic curve has two peaks at about 1.03 and 1.56 V, corresponding to the oxidation of Fe to Fe^2+^ and Fe^3+^, respectively. The anode curves also show a similar profile from the second cycle. The broad peak at 1.56 V can be related to the reversible oxidation of the metallic Fe phase.

Figure 4b shows the specific capacity and voltage curves of the first three and the 100th cycles of the Fe_3_O_4_@C composite at 1000 mA·g^−1^. It can be seen that the charge/discharge plateaus match with the redox peaks from the corresponding CV curves. The lithiation and delithiation capacities of the Fe_3_O_4_@C sample in the first cycle are 978.7 and 547.3 mAh·g^−1^, respectively, and the coulombic efficiency is 55.9%. In addition, the shapes of the charge and discharge curves for the 100th cycle is in good agreement with the first three cycles, indicating that the Fe_3_O_4_@C sample has good potential structural stability in the long-term.

**Figure 5:**
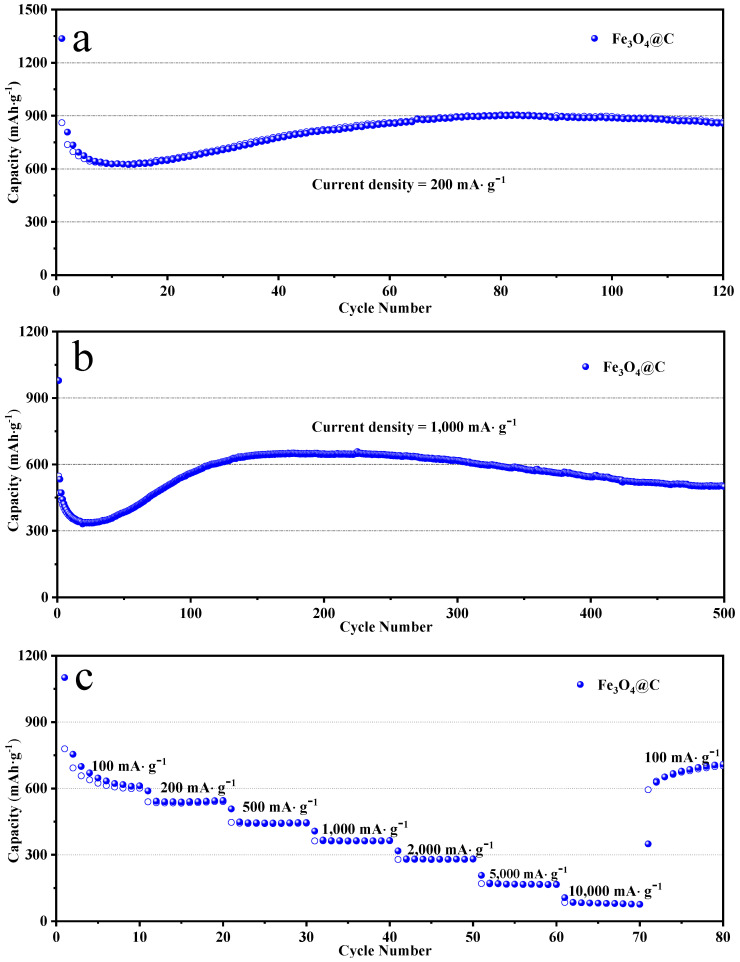
Low-current cycling performance, (**a**) high-current cycling performance, (**b**) and rate capability (**c**) of the Fe_3_O_4_@C sample.

Figure 5a shows the cycling performance of the Fe_3_O_4_@C composite at a low current rate of 200 mA·g^−1^. In the first cycle, this sample delivers the charge/discharge specific capacities of 1336.1 and 860.8 mAh·g^−1^, respectively, with a corresponding coulombic efficiency of 64.4%. After a gradual activation process in the following few cycles, stable rising capacities have been witnessed, and a high reversible capacity of 865.0 mAh·g^−1^ after 120 cycles can be observed for this sample. Moreover, as shown in Figure 5b, the Fe_3_O_4_@C composite shows an initial discharge specific capacity of 978.7 mAh·g^−1^ at a high current rate of 1000 mA·g^−1^, which can still provide a capacity of 500.2 mAh·g^−1^ after 500 cycles. As can be seen from Figure 5c, the Fe_3_O_4_@C composite also exhibit good rate characteristics. As the current rate increases, the specific capacity of the sample decreases accordingly. At a current rate of 100, 200, 500, 1000, 2000, 5000, and 10,000 mA·g^−1^, the average reversible capacities of the sample were 601.2, 540.4, 443.0, 363.5, 280.6, 166.3, and 76.1 mAh·g^−1^, respectively. The current rate was then reduced again to 100 mA·g^−1^, and the specific capacity increased and remained stable, with an average capacity of about 702.6 mAh·g^−1^.

**Figure 6:**
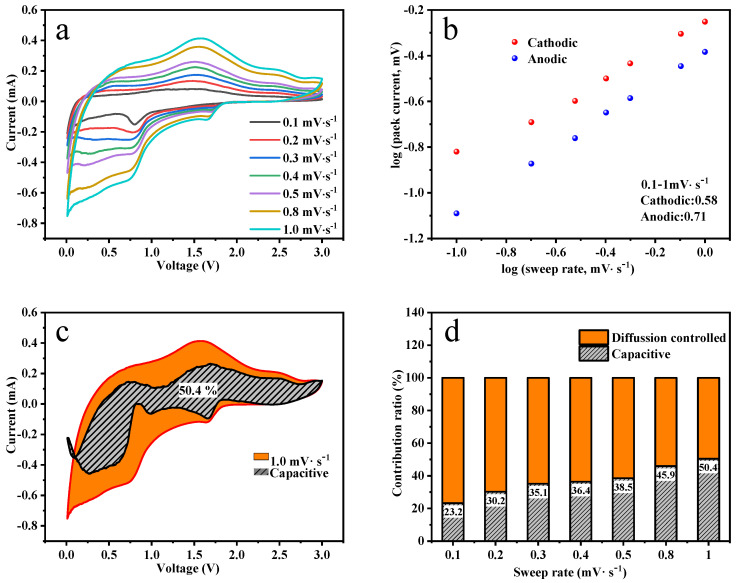
CV curves at different scanning rates from 0.1 to 1.0 mV s^−1^ (**a**), the log (i)-log (v) plot (**b**); the calculated pseudocapacitive contribution diagram at the highest scanning voltage rate of 1.0 mV s^−1^ (**c**) and full pseudocapacitive contribution ratios at different scanning rates (**d**) of the Fe_3_O_4_@C composite before cycling.

The contributions of pseudocapacitance and battery behavior to the lithium-ion storage performance of the Fe_3_O_4_@C composite are analyzed. Figure 6a depicts the CV curves between the gradient sweep velocity of 0.1–1.0 mV·s^−1^. The relationship between peak current and sweep speed can be calculated from Equation (1) below [34].

i = av^b^ (1)

Here, the log(i)–log(v) values calculated from the oxidized and reduced portions in the CV curves above are shown in Figure 6b. A clear linear relationship can be seen, and the b values calculated from the corresponding slopes are 0.58 (cathode peak) and 0.71 (anode peak), respectively, which indicates that pseudocapacitance behavior and battery behavior have a joint contribution to the lithium-ion storage in the Fe_3_O_4_@C electrode.

Based on Equation (2), it can further quantify the contribution ratio of battery behavior and pseudocapacitance behavior.

i(V) = k_1_v + k_2_v^1/2^ (2)

where i(V), k_1_v, and k_2_v^1/2^ represent the current at a fixed potential, capacitive-controlled current and diffusion-controlled current, respectively. Subsequently, the capacitive part can be calculated by fitting k_1_ and k_2_ at a fixed potential. As shown in Figure 6c, at 1.0 mV·s^−1^, the capacitance contribution shown in the dark region accounts for 50.4 % of the total capacity. As the scan rate increases from 0.1 mV·s^−1^ to 1.0 mV·s^−1^, in Figure 6d, it can be seen that the capacitance contribution ratio ranges from 23.2%, 30.2%, 35.1%, 36.4%, 38.5%, 45.9%, to 50.4%. Obviously, the capacitance contribution increases as the scan voltage rate increases, which brings in accelerated lithium-ion storage behavior with little structural degradation of the active Fe_3_O_4_@C material, confirming the high rate performance and long cycling stability.

**Figure 7:**
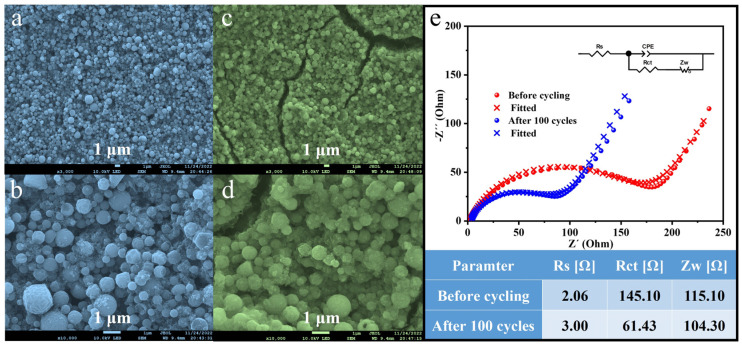
FESEM images of the Fe_3_O_4_@C electrode before cycling (**a**,**b**) and after 100 cycles (**c**,**d**) under the corresponding magnifications; Nyquist plots of the Fe_3_O_4_@C electrode before cycling and after 100 cycles with the equivalent circuit and parameters obtained by fitting the EIS data (**e**).

The structural evolution of the anode prepared with Fe_3_O_4_@C as the electrochemically active material before and after 100 cycles is studied. For the FESEM images before cycling in Figure 7a,b, submicron sized Fe_3_O_4_@C spheres can be clearly identified, and space can also be seen between the individual Fe_3_O_4_@C samples. After 100 cycles, the electrode surface becomes slightly cracked due to the dissembling of the coin cells and further washing and drying of the electrode in Figure 7c. While from Figure 7d, the surface of the Fe_3_O_4_@C spheres becomes slightly roughened due to the generation of SEI film. In addition, no obvious morphological change can be found, demonstrating the good structure stability of the Fe_3_O_4_@C composite.

The Nyquist plots of the Fe_3_O_4_@C electrode before cycling and after 100 cycles are shown in Figure 7e. The high frequency region usually contains one or two semicircles corresponding to the formation of SEI (Rf) and charge transfer resistance (Rct), respectively, while the low frequency region contains only an inclined straight line representing the Warburg impedance (Zw) of lithium-ion diffusion [35]. By using ZView software, the fitted parameters of the two curves are calculated based on the inset equivalent circuit. Obviously, the Rct of the Fe_3_O_4_@C composite electrode is 61.43 Ω after 100 cycles, which is significantly lower than the 165 Ω before cycling. This result demonstrates that the Fe_3_O_4_@C electrode could provide largely improved charge transfer conditions after gradual electrochemical activation.

## 4. Conclusions

In sum, CS molecules and FeCl_3_ salt were selected as the raw materials for the direct synthesis a nitrogen-doped carbon matrix supported Fe_3_O_4_ nanoparticle composite (Fe_3_O_4_@C) via a one-step spray pyrolysis treatment. Protected by inert gas, the homogeneously mixed Fe^3+^ ions and CS molecules are in situ transformed to Fe_3_O_4_ nanoparticles and spherical pyrolytic carbon coating domains, respectively. Moreover, the CS derived submicron sized carbon framework exhibits a pomegranate configuration with small sized Fe_3_O_4_ nanoparticles homogenously dispersed. Therefore, a favorable conductive network and buffering space have been created in the Fe_3_O_4_@C composite. When used as an anode electrochemical active material, the Fe_3_O_4_@C composite exhibits inspiring lithium-ion storage capabilities, which maintains a high specific capacity of 865.0 mAh·g^−1^ at a low current density of 200 mA·g^−1^ after 120 cycles and a stable reversible capacity of 500.1 mAh·g^−1^ at a high current density of 1000 mA·g^−1^ after 500 cycles. The robust structural stability and hybrid lithium-ion storage mechanism are also beneficial for elevating the lithium-ion storage performances of the Fe_3_O_4_@C composite.

## Data Availability

Not applicable.
